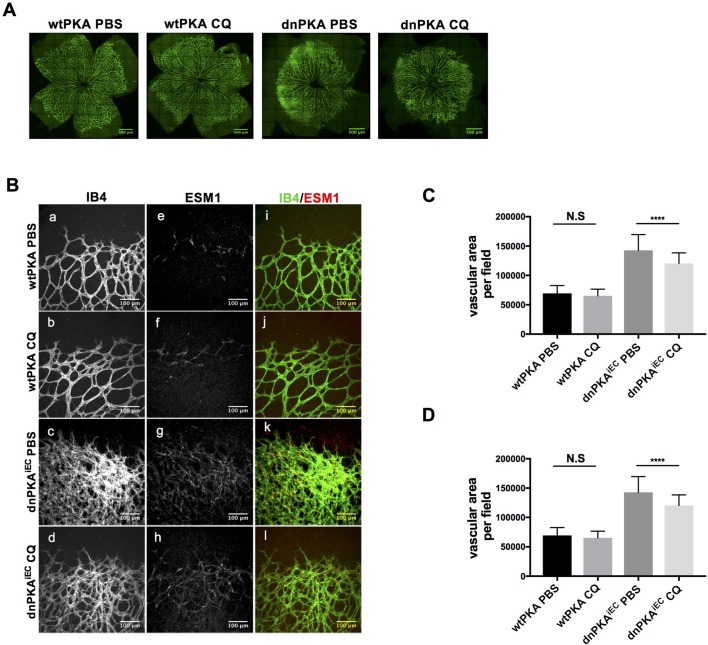# Correction: Endothelial PKA activity regulates angiogenesis by limiting autophagy through phosphorylation of ATG16L1

**DOI:** 10.7554/eLife.58195

**Published:** 2020-04-27

**Authors:** Xiaocheng Zhao, Pavel Nedvetsky, Fabio Stanchi, Anne-Clemence Vion, Oliver Popp, Kerstin Zühlke, Gunnar Dittmar, Enno Klussmann, Holger Gerhardt

Zhao X, Nedvetsky P, Stanchi F, Vion AC, Popp O, Zühlke K, Dittmar G, Klussmann E, Gerhardt H. 2019. Endothelial PKA activity regulates angiogenesis by limiting autophagy through phosphorylation of ATG16L1. *eLife* **8**:e46380. doi: 10.7554/eLife.46380.

Published 03, October 2019

We have discovered a panel duplication in Figure 4 supplement 1C and D. It appears that during reorganisation of this Figure supplement in the course of revisions, panel 1C was duplicated as 1D. The correct panel was present in the original version of the figure, showing the quantification of ESM1 positive area in 1D. We further discovered a small typesetting error in the method section which read ml/min instead of µl/min. We would like to correct these errors as follows. The corrections do not affect data or conclusions.

The corrected text (change is underlined):

Five microliters of the sample was loaded on a nano-LC column (0.074 × 250 mm, 3 mm Reprosil C18; Dr. Maisch) and separated on a 155 min gradient (4%–76% acetonitrile) at a flow rate of 0.25 μl/min and ionized using a Proxeon ion source. Mass spectrometric acquisition was done at a resolution of 60,000 with a scan range of 200–1,700 m/z in FTMS mode selecting the top 20 peaks for collision-induced dissociation fragmentation.

The corrected Figure 4 supplement 1 is shown here:

**Figure 1:**
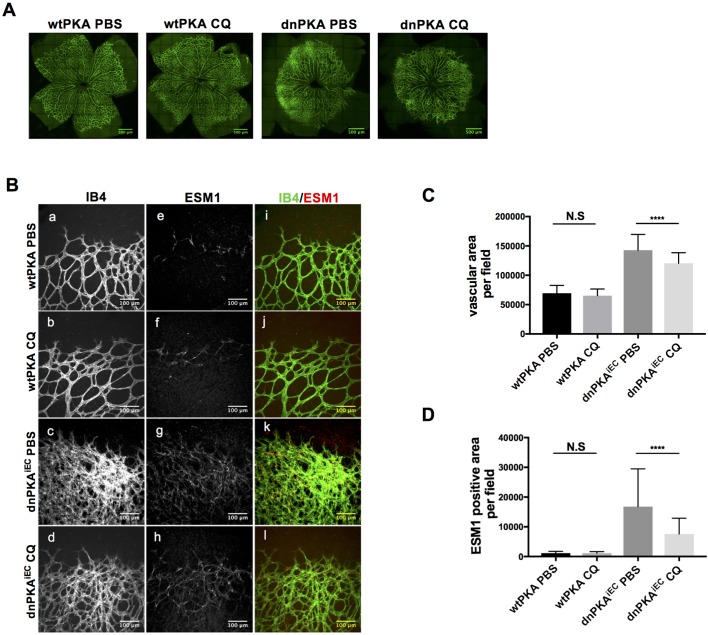


The originally published Figure 4 supplement 1is also shown for reference:

**Figure 2:**