# Supplementary material for: Endothelial PKA activity regulates angiogenesis by limiting autophagy through phosphorylation of ATG16L1
Source: eLife. 2019 Oct 3;8:e46380. doi: 10.7554/eLife.46380 (PMC6797479; doi:10.7554/eLife.46380)
Supplement: Table 1—source data 1. [file elife-46380-table1-data1.docx]

Table 1-source data 1

| **Uniprot** | **Protein.names** | **Gene.names** | **Peptides** | **Log ratio AS/WT** | |
| --- | --- | --- | --- | --- | --- |
|  |  |  |  | **experiment1** | **experiment2** |
| Q6AI12 | Ankyrin repeat domain-containing protein 40 | ANKRD40 | 9 | 10 | 10 |
| Q6P6C2 | RNA demethylase ALKBH5 | ALKBH5 | 6 | 10 | 10 |
| Q9NRY4 | Rho GTPase-activating protein 35 | ARHGAP35 | 9 | 10 | 10 |
| E7EVC7 | Autophagy-related protein 16-1 | ATG16L1 | 8 | 10 | 10 |
| J3KPC8 | Serine/threonine-protein kinase SIK3 | SIK3;KIAA0999 | 5 | 10 | 10 |
| A1X283 | SH3 and PX domain-containing protein 2B | SH3PXD2B | 4 | 10 | 10 |
| Q8IWZ8 | SURP and G-patch domain-containing protein 1 | SUGP1 | 5 | 10 | 10 |
| Q9UJX5 | Anaphase-promoting complex subunit 4 | ANAPC4 | 5 | 10 | 10 |
| O43719 | HIV Tat-specific factor 1 | HTATSF1 | 4 | 10 | 10 |
| O95644-5 | Nuclear factor of activated T-cells, cytoplasmic 1 | NFATC1 | 5 | 10 | 10 |
| G8JLI6 | Prolyl 3-hydroxylase 3 | LEPREL2 | 3 | 10 | 10 |
| F8W781 | Zinc finger CCCH domain-containing protein 13 | ZC3H13 | 3 | 10 | 10 |
| Q9Y4G8 | Rap guanine nucleotide exchange factor 2 | RAPGEF2 | 21 | 10 | 6,17455504 |
| J3KNX9 | Unconventional myosin-XVIIIa | MYO18A | 10 | 10 | 4,43208178 |
| Q15111 | Inactive phospholipase C-like protein 1;Phosphoinositide phospholipase C | PLCL1 | 10 | 10 | 3,32188704 |
| Q9ULI2 | Beta-citrylglutamate synthase B | RIMKLB | 4 | 10 | 2,71571603 |
| E9PG19 | FCH and double SH3 domains protein 2 | FCHSD2 | 4 | 10 | 2,36224567 |
| H7C4H2 | Signal recognition particle receptor subunit beta | SRPRB | 2 | 10 | 1,9069161 |
| E9PFQ4 | Pleckstrin homology-like domain family B member 2 | PHLDB2 | 7 | 10 | 1,74771272 |
| Q15637-4 | Splicing factor 1 | SF1 | 5 | 10 | 1,49993486 |
| Q2M1Z3 | Rho GTPase-activating protein 31 | ARHGAP31 | 5 | 10 | 1,33250391 |
| Q96ST2-2 | Protein IWS1 homolog | IWS1 | 3 | 10 | 1,26770303 |
| P31323 | cAMP-dependent protein kinase type II-beta regulatory subunit | PRKAR2B | 19 | 7,05077105 | 5,33509437 |
| Q00537 | Cyclin-dependent kinase 17 | CDK17 | 31 | 6,37867381 | 6,39216838 |
| Q9BZL4 | Protein phosphatase 1 regulatory subunit 12C | PPP1R12C | 21 | 6,04440274 | 7,30701515 |
| O14974 | Protein phosphatase 1 regulatory subunit 12A | PPP1R12A | 26 | 5,72796034 | 7,12654716 |
| P13861 | cAMP-dependent protein kinase type II-alpha regulatory subunit | PRKAR2A | 24 | 5,42841998 | 5,04010629 |
| Q86UU1-2 | Pleckstrin homology-like domain family B member 1 | PHLDB1 | 19 | 5,4105243 | 4,10782285 |
| Q9BYB0 | SH3 and multiple ankyrin repeat domains protein 3 | SHANK3 | 32 | 5,26591421 | 5,6389181 |
| O15056 | Synaptojanin-2 | SYNJ2 | 13 | 4,64022655 | 4,46069701 |
| J3KSW8 | Myosin phosphatase Rho-interacting protein | MPRIP | 18 | 4,61398477 | 5,61155414 |
| Q14185 | Dedicator of cytokinesis protein 1 | DOCK1 | 34 | 4,50413426 | 3,18515106 |
| Q9HD67 | Unconventional myosin-X | MYO10 | 39 | 4,13973415 | 3,21827463 |
| Q9H1A4 | Anaphase-promoting complex subunit 1 | ANAPC1 | 10 | 3,78601972 | 1,64967037 |
| Q99996-3 | A-kinase anchor protein 9 | AKAP9 | 27 | 3,56917194 | 2,96594568 |
| Q14980-2 | Nuclear mitotic apparatus protein 1 | NUMA1 | 61 | 3,45625969 | 4,47466712 |
| P28715 | DNA repair protein complementing XP-G cells | ERCC5;BIVM-ERCC5 | 8 | 3,3571826 | 4,09305592 |
| P12270 | Nucleoprotein TPR | TPR | 104 | 3,3333472 | 4,04477536 |
| O75116 | Rho-associated protein kinase 2 | ROCK2 | 29 | 3,27701864 | 3,08277835 |
| P46013 | Antigen KI-67 | MKI67 | 6 | 2,9817545 | 1,79835037 |
| Q96PE2 | Rho guanine nucleotide exchange factor 17 | ARHGEF17 | 7 | 2,94191318 | 1,08919317 |
| Q9BXF6 | Rab11 family-interacting protein 5 | RAB11FIP5 | 6 | 2,72040086 | 3,00005811 |
| Q99661-2 | Kinesin-like protein KIF2C | KIF2C | 13 | 2,63985171 | 2,81061048 |
| S4R313 | Palmdelphin | PALMD | 5 | 2,54693311 | 2,1388952 |
| Q9H1Y0 | Autophagy protein 5 | ATG5 | 5 | 2,46901177 | 2,20268577 |
| F5H5C2 | Nuclear pore complex protein Nup133 | NUP133 | 23 | 2,42965373 | 2,20028491 |
| Q68EM7-6 | Rho GTPase-activating protein 17 | ARHGAP17 | 13 | 2,35269231 | 2,35930375 |
| Q9NQT8 | Kinesin-like protein KIF13B | KIF13B | 9 | 2,34172497 | 1,68784955 |
| Q14149 | MORC family CW-type zinc finger protein 3 | MORC3 | 16 | 2,32261659 | 2,52954027 |
| Q08AD1-2 | Calmodulin-regulated spectrin-associated protein 2 | CAMSAP2 | 8 | 2,31241733 | 10 |
| P30260 | Cell division cycle protein 27 homolog | CDC27 | 23 | 2,30625179 | 2,25248961 |
| Q9Y2J2-2 | Band 4.1-like protein 3;Band 4.1-like protein 3, N-terminally processed | EPB41L3 | 31 | 2,25892933 | 2,19944372 |
| F5H1U9 | Multiple PDZ domain protein | MPDZ | 6 | 2,19513391 | 2,55367573 |
| H3BLZ8 | Probable ATP-dependent RNA helicase DDX17 | DDX17 | 32 | 2,1394526 | 1,36379114 |
| F5H0F9 | Anaphase-promoting complex subunit 5 | ANAPC5 | 9 | 2,09716742 | 1,27582571 |
| Q9Y2D5 | A-kinase anchor protein 2 | AKAP2 | 20 | 2,09090469 | 3,03576783 |
| Q9UJX3-2 | Anaphase-promoting complex subunit 7 | ANAPC7 | 10 | 2,08490939 | 2,29200131 |
| Q9P219 | Protein Daple | CCDC88C | 15 | 2,0621253 | 3,02063473 |
| Q5T8C6 | Cell division cycle protein 16 homolog | CDC16 | 10 | 1,93857345 | 1,76200726 |
| E7EVX8 | U4/U6 small nuclear ribonucleoprotein Prp31 | PRPF31 | 9 | 1,850312 | 1,84552738 |
| P23526 | Adenosylhomocysteinase | AHCY | 8 | 1,77811028 | 1,26775316 |
| E7EWQ5 | Microtubule-associated serine/threonine-protein kinase 4 | MAST4 | 13 | 1,69313999 | 1,01367536 |
| Q9P0K7-4 | Ankycorbin | RAI14 | 27 | 1,67510459 | 2,15586213 |
| P29279 | Connective tissue growth factor | CTGF | 4 | 1,62418869 | 1,08675491 |
| F8VV52 | CCR4-NOT transcription complex subunit 2 | CNOT2 | 5 | 1,60990968 | 1,02123394 |
| O75175-3 | CCR4-NOT transcription complex subunit 3 | CNOT3 | 5 | 1,60311775 | 1,46191933 |
| O00139-2 | Kinesin-like protein KIF2A | KIF2A | 8 | 1,57140421 | 1,44078888 |
| P25205 | DNA replication licensing factor MCM3 | MCM3 | 26 | 1,53099827 | 1,27098388 |
| O60333-3 | Kinesin-like protein KIF1B;Kinesin-like protein KIF1A | KIF1B;KIF1A | 8 | 1,52559355 | 10 |
| Q16512 | Serine/threonine-protein kinase N1 | PKN1 | 9 | 1,47301073 | 1,87711639 |
| Q9BZK7 | F-box-like/WD repeat-containing protein TBL1XR1 | TBL1XR1 | 10 | 1,45606041 | 2,32867324 |
| O14828 | Secretory carrier-associated membrane protein 3 | SCAMP3 | 6 | 1,41148696 | 1,05840205 |
| Q08499-6 | cAMP-specific 3,5-cyclic phosphodiesterase 4D | PDE4D | 6 | 1,40991488 | 1,63183699 |
| A8MXP9 | Matrin-3 | MATR3 | 28 | 1,39072068 | 1,37496331 |
| P53396-2 | ATP-citrate synthase | ACLY | 36 | 1,37386941 | 1,00149978 |
| P50552 | Vasodilator-stimulated phosphoprotein | VASP | 14 | 1,36669933 | 2,73634493 |
| Q8N3V7-2 | Synaptopodin | SYNPO | 12 | 1,36190044 | 2,58547274 |
| P61981 | 14-3-3 protein gamma;14-3-3 protein gamma, N-terminally processed | YWHAG | 8 | 1,36086566 | 1,57610229 |
| C9JA28 | Translocon-associated protein subunit gamma | SSR3 | 1 | 1,32037799 | 1,36697094 |
| Q5UIP0-2 | Telomere-associated protein RIF1 | RIF1 | 61 | 1,30791501 | 1,04266954 |
| Q9UJX2-3 | Cell division cycle protein 23 homolog | CDC23 | 8 | 1,28514428 | 1,66294981 |
| Q9UNF1 | Melanoma-associated antigen D2 | MAGED2 | 16 | 1,28222114 | 1,18348318 |
| O95456-2 | Proteasome assembly chaperone 1 | PSMG1 | 3 | 1,26853447 | 3,11591507 |
| Q9H2D6-5 | TRIO and F-actin-binding protein | TRIOBP | 22 | 1,25879668 | 1,63620678 |
| J3QRS9 | BUB3-interacting and GLEBS motif-containing protein ZNF207 | ZNF207 | 6 | 1,2171795 | 1,16059002 |
| Q15149-6 | Plectin | PLEC | 92 | 1,21549706 | 1,03559459 |
| Q9NSD9 | Phenylalanine--tRNA ligase beta subunit | FARSB | 5 | 1,14392912 | 10 |
| Q01082 | Spectrin beta chain, non-erythrocytic 1 | SPTBN1 | 67 | 1,13754361 | 1,61784987 |
| O94885 | SAM and SH3 domain-containing protein 1 | SASH1 | 10 | 1,13593257 | 1,01717758 |
| Q32P28 | Prolyl 3-hydroxylase 1 | LEPRE1 | 9 | 1,11525049 | 1,76253481 |
| P57740 | Nuclear pore complex protein Nup107 | NUP107 | 18 | 1,11522237 | 1,20865718 |
| G3V2E7 | Kinesin light chain 1 | KLC1 | 20 | 1,10212078 | 1,20673135 |
| P33176 | Kinesin-1 heavy chain | KIF5B | 43 | 1,09718553 | 1,29668785 |
| P37268 | Squalene synthase | FDFT1 | 7 | 1,07209501 | 1,63214806 |
| O60271-4 | C-Jun-amino-terminal kinase-interacting protein 4 | SPAG9 | 6 | 1,03259304 | 1,04202752 |
| Q5SYE7-2 | NHS-like protein 1 | NHSL1 | 6 | 1,01211229 | 2,07660272 |
| Q9C0C2 | 182 kDa tankyrase-1-binding protein | TNKS1BP1 | 48 | 1,00029269 | 1,39896147 |
