# Supplementary material for: Endothelial PKA activity regulates angiogenesis by limiting autophagy through phosphorylation of ATG16L1
Source: eLife. 2019 Oct 3;8:e46380. doi: 10.7554/eLife.46380 (PMC6797479; doi:10.7554/eLife.46380)
Supplement: Supplementary file 1. [file elife-46380-supp1.docx]

Figure 2 supplement 1 2A spots map

ATG16L1α SPOTS MAP

**Spot Position MolWeight Sequence**

1 A 1 3067.600 M-S-S-G-L-R-A-A-D-F-P-R-W-K-R-H-I-S-E-Q-L-R-R-R-D

2 B 1 3273.793 R-A-A-D-F-P-R-W-K-R-H-I-S-E-Q-L-R-R-R-D-R-L-Q-R-Q

3 C 1 3302.797 P-R-W-K-R-H-I-S-E-Q-L-R-R-R-D-R-L-Q-R-Q-A-F-E-E-I

4 D 1 3210.701 H-I-S-E-Q-L-R-R-R-D-R-L-Q-R-Q-A-F-E-E-I-I-L-Q-Y-N

5 E 1 3227.826 L-R-R-R-D-R-L-Q-R-Q-A-F-E-E-I-I-L-Q-Y-N-K-L-L-E-K

6 F 1 3070.645 R-L-Q-R-Q-A-F-E-E-I-I-L-Q-Y-N-K-L-L-E-K-S-D-L-H-S

7 G 1 2928.585 A-F-E-E-I-I-L-Q-Y-N-K-L-L-E-K-S-D-L-H-S-V-L-A-Q-K

8 H 1 2908.628 I-L-Q-Y-N-K-L-L-E-K-S-D-L-H-S-V-L-A-Q-K-L-Q-A-E-K

9 I 1 2839.545 K-L-L-E-K-S-D-L-H-S-V-L-A-Q-K-L-Q-A-E-K-H-D-V-P-N

10 J 1 2850.463 S-D-L-H-S-V-L-A-Q-K-L-Q-A-E-K-H-D-V-P-N-R-H-E-I-S

11 K 1 2774.410 V-L-A-Q-K-L-Q-A-E-K-H-D-V-P-N-R-H-E-I-S-P-G-H-D-G

12 L 1 2865.307 L-Q-A-E-K-H-D-V-P-N-R-H-E-I-S-P-G-H-D-G-T-W-N-D-N

13 M 1 2925.274 H-D-V-P-N-R-H-E-I-S-P-G-H-D-G-T-W-N-D-N-Q-L-Q-E-M

14 N 1 2944.389 R-H-E-I-S-P-G-H-D-G-T-W-N-D-N-Q-L-Q-E-M-A-Q-L-R-I

15 O 1 2973.368 P-G-H-D-G-T-W-N-D-N-Q-L-Q-E-M-A-Q-L-R-I-K-H-Q-E-E

16 P 1 3103.503 T-W-N-D-N-Q-L-Q-E-M-A-Q-L-R-I-K-H-Q-E-E-L-T-E-L-H

17 Q 1 3071.619 Q-L-Q-E-M-A-Q-L-R-I-K-H-Q-E-E-L-T-E-L-H-K-K-R-G-E

18 R 1 2966.667 A-Q-L-R-I-K-H-Q-E-E-L-T-E-L-H-K-K-R-G-E-L-A-Q-L-V

19 S 1 2954.583 K-H-Q-E-E-L-T-E-L-H-K-K-R-G-E-L-A-Q-L-V-I-D-L-N-N

20 T 1 2974.639 L-T-E-L-H-K-K-R-G-E-L-A-Q-L-V-I-D-L-N-N-Q-M-Q-R-K

21 A 2 3040.591 K-K-R-G-E-L-A-Q-L-V-I-D-L-N-N-Q-M-Q-R-K-D-R-E-M-Q

22 B 2 3015.494 L-A-Q-L-V-I-D-L-N-N-Q-M-Q-R-K-D-R-E-M-Q-M-N-E-A-K

23 C 2 3020.419 I-D-L-N-N-Q-M-Q-R-K-D-R-E-M-Q-M-N-E-A-K-I-A-E-C-L

24 D 2 2995.387 Q-M-Q-R-K-D-R-E-M-Q-M-N-E-A-K-I-A-E-C-L-Q-T-I-S-D

25 E 2 2899.260 D-R-E-M-Q-M-N-E-A-K-I-A-E-C-L-Q-T-I-S-D-L-E-T-E-C

26 F 2 2838.334 M-N-E-A-K-I-A-E-C-L-Q-T-I-S-D-L-E-T-E-C-L-D-L-R-T

27 G 2 2837.375 I-A-E-C-L-Q-T-I-S-D-L-E-T-E-C-L-D-L-R-T-K-L-C-D-L

28 H 2 2906.400 Q-T-I-S-D-L-E-T-E-C-L-D-L-R-T-K-L-C-D-L-E-R-A-N-Q

29 I 2 2948.447 L-E-T-E-C-L-D-L-R-T-K-L-C-D-L-E-R-A-N-Q-T-L-K-D-E

30 J 2 2963.491 L-D-L-R-T-K-L-C-D-L-E-R-A-N-Q-T-L-K-D-E-Y-D-A-L-Q

31 K 2 2898.432 K-L-C-D-L-E-R-A-N-Q-T-L-K-D-E-Y-D-A-L-Q-I-T-F-T-A

32 L 2 2866.460 E-R-A-N-Q-T-L-K-D-E-Y-D-A-L-Q-I-T-F-T-A-L-E-G-K-L

33 M 2 2883.512 T-L-K-D-E-Y-D-A-L-Q-I-T-F-T-A-L-E-G-K-L-R-K-T-T-E

34 N 2 2910.486 Y-D-A-L-Q-I-T-F-T-A-L-E-G-K-L-R-K-T-T-E-E-N-Q-E-L

35 O 2 2993.553 I-T-F-T-A-L-E-G-K-L-R-K-T-T-E-E-N-Q-E-L-V-T-R-W-M

36 P 2 2987.539 L-E-G-K-L-R-K-T-T-E-E-N-Q-E-L-V-T-R-W-M-A-E-K-A-Q

37 Q 2 3030.519 R-K-T-T-E-E-N-Q-E-L-V-T-R-W-M-A-E-K-A-Q-E-A-N-R-L

38 R 2 2972.394 E-N-Q-E-L-V-T-R-W-M-A-E-K-A-Q-E-A-N-R-L-N-A-E-N-E

39 S 2 3001.479 V-T-R-W-M-A-E-K-A-Q-E-A-N-R-L-N-A-E-N-E-K-D-S-R-R

40 T 2 2952.524 A-E-K-A-Q-E-A-N-R-L-N-A-E-N-E-K-D-S-R-R-R-Q-A-R-L

41 A 3 2994.571 E-A-N-R-L-N-A-E-N-E-K-D-S-R-R-R-Q-A-R-L-Q-K-E-L-A

42 B 3 2939.517 N-A-E-N-E-K-D-S-R-R-R-Q-A-R-L-Q-K-E-L-A-E-A-A-K-E

43 C 3 2917.610 K-D-S-R-R-R-Q-A-R-L-Q-K-E-L-A-E-A-A-K-E-P-L-P-V-E

44 D 3 2861.477 R-Q-A-R-L-Q-K-E-L-A-E-A-A-K-E-P-L-P-V-E-Q-D-D-D-I

45 E 3 2792.386 Q-K-E-L-A-E-A-A-K-E-P-L-P-V-E-Q-D-D-D-I-E-V-I-V-D

46 F 3 2792.277 E-A-A-K-E-P-L-P-V-E-Q-D-D-D-I-E-V-I-V-D-E-T-S-D-H

47 G 3 2811.235 P-L-P-V-E-Q-D-D-D-I-E-V-I-V-D-E-T-S-D-H-T-E-E-T-S

48 H 3 2812.278 Q-D-D-D-I-E-V-I-V-D-E-T-S-D-H-T-E-E-T-S-P-V-R-A-I

49 I 3 2712.309 E-V-I-V-D-E-T-S-D-H-T-E-E-T-S-P-V-R-A-I-S-R-A-A-T

50 J 3 2742.353 E-T-S-D-H-T-E-E-T-S-P-V-R-A-I-S-R-A-A-T-R-R-S-V-S

51 K 3 2700.420 T-E-E-T-S-P-V-R-A-I-S-R-A-A-T-R-R-S-V-S-S-F-P-V-P

52 L 3 2724.431 P-V-R-A-I-S-R-A-A-T-R-R-S-V-S-S-F-P-V-P-Q-D-N-V-D

53 M 3 2667.300 S-R-A-A-T-R-R-S-V-S-S-F-P-V-P-Q-D-N-V-D-T-H-P-G-S

54 N 3 2750.374 R-R-S-V-S-S-F-P-V-P-Q-D-N-V-D-T-H-P-G-S-G-K-E-V-R

55 O 3 2604.282 S-F-P-V-P-Q-D-N-V-D-T-H-P-G-S-G-K-E-V-R-V-P-A-T-A

56 P 3 2654.265 Q-D-N-V-D-T-H-P-G-S-G-K-E-V-R-V-P-A-T-A-L-C-V-F-D

57 Q 3 2592.228 T-H-P-G-S-G-K-E-V-R-V-P-A-T-A-L-C-V-F-D-A-H-D-G-E

58 R 3 2624.291 G-K-E-V-R-V-P-A-T-A-L-C-V-F-D-A-H-D-G-E-V-N-A-V-Q

59 S 3 2530.169 V-P-A-T-A-L-C-V-F-D-A-H-D-G-E-V-N-A-V-Q-F-S-P-G-S

60 T 3 2645.280 L-C-V-F-D-A-H-D-G-E-V-N-A-V-Q-F-S-P-G-S-R-L-L-A-T

61 A 4 2584.234 A-H-D-G-E-V-N-A-V-Q-F-S-P-G-S-R-L-L-A-T-G-G-M-D-R

62 B 4 2757.475 V-N-A-V-Q-F-S-P-G-S-R-L-L-A-T-G-G-M-D-R-R-V-K-L-W

63 C 4 2807.443 F-S-P-G-S-R-L-L-A-T-G-G-M-D-R-R-V-K-L-W-E-V-F-G-E

64 D 4 2967.546 R-L-L-A-T-G-G-M-D-R-R-V-K-L-W-E-V-F-G-E-K-C-E-F-K

65 E 4 2814.383 G-G-M-D-R-R-V-K-L-W-E-V-F-G-E-K-C-E-F-K-G-S-L-S-G

66 F 4 2740.390 R-V-K-L-W-E-V-F-G-E-K-C-E-F-K-G-S-L-S-G-S-N-A-G-I

67 G 4 2635.237 E-V-F-G-E-K-C-E-F-K-G-S-L-S-G-S-N-A-G-I-T-S-I-E-F

68 H 4 2491.143 K-C-E-F-K-G-S-L-S-G-S-N-A-G-I-T-S-I-E-F-D-S-A-G-S

69 I 4 2387.138 G-S-L-S-G-S-N-A-G-I-T-S-I-E-F-D-S-A-G-S-Y-L-L-A-A

70 J 4 2520.155 S-N-A-G-I-T-S-I-E-F-D-S-A-G-S-Y-L-L-A-A-S-N-D-F-A

71 K 4 2721.281 T-S-I-E-F-D-S-A-G-S-Y-L-L-A-A-S-N-D-F-A-S-R-I-W-T

72 L 4 2792.293 D-S-A-G-S-Y-L-L-A-A-S-N-D-F-A-S-R-I-W-T-V-D-D-Y-R

73 M 4 2995.519 Y-L-L-A-A-S-N-D-F-A-S-R-I-W-T-V-D-D-Y-R-L-R-H-T-L

74 N 4 2903.395 S-N-D-F-A-S-R-I-W-T-V-D-D-Y-R-L-R-H-T-L-T-G-H-S-G

75 O 4 2867.504 S-R-I-W-T-V-D-D-Y-R-L-R-H-T-L-T-G-H-S-G-K-V-L-S-A

76 P 4 2840.519 V-D-D-Y-R-L-R-H-T-L-T-G-H-S-G-K-V-L-S-A-K-F-L-L-D

77 Q 4 2745.566 L-R-H-T-L-T-G-H-S-G-K-V-L-S-A-K-F-L-L-D-N-A-R-I-V

78 R 4 2608.361 T-G-H-S-G-K-V-L-S-A-K-F-L-L-D-N-A-R-I-V-S-G-S-H-D

79 S 4 2780.591 K-V-L-S-A-K-F-L-L-D-N-A-R-I-V-S-G-S-H-D-R-T-L-K-L

80 T 4 2939.598 K-F-L-L-D-N-A-R-I-V-S-G-S-H-D-R-T-L-K-L-W-D-L-R-S

81 A 5 2894.591 N-A-R-I-V-S-G-S-H-D-R-T-L-K-L-W-D-L-R-S-K-V-C-I-K

82 B 5 2816.501 S-G-S-H-D-R-T-L-K-L-W-D-L-R-S-K-V-C-I-K-T-V-F-A-G

83 C 5 2839.473 R-T-L-K-L-W-D-L-R-S-K-V-C-I-K-T-V-F-A-G-S-S-C-N-D

84 D 5 2773.313 W-D-L-R-S-K-V-C-I-K-T-V-F-A-G-S-S-C-N-D-I-V-C-T-E

85 E 5 2664.198 K-V-C-I-K-T-V-F-A-G-S-S-C-N-D-I-V-C-T-E-Q-C-V-M-S

86 F 5 2677.117 T-V-F-A-G-S-S-C-N-D-I-V-C-T-E-Q-C-V-M-S-G-H-F-D-K

87 G 5 2932.302 S-S-C-N-D-I-V-C-T-E-Q-C-V-M-S-G-H-F-D-K-K-I-R-F-W

88 H 5 3026.445 I-V-C-T-E-Q-C-V-M-S-G-H-F-D-K-K-I-R-F-W-D-I-R-S-E

89 I 5 3065.522 Q-C-V-M-S-G-H-F-D-K-K-I-R-F-W-D-I-R-S-E-S-I-V-R-E

90 J 5 3060.586 G-H-F-D-K-K-I-R-F-W-D-I-R-S-E-S-I-V-R-E-M-E-L-L-G

91 K 5 3002.663 K-I-R-F-W-D-I-R-S-E-S-I-V-R-E-M-E-L-L-G-K-I-T-A-L

92 L 5 2840.484 D-I-R-S-E-S-I-V-R-E-M-E-L-L-G-K-I-T-A-L-D-L-N-P-E

93 M 5 2852.557 S-I-V-R-E-M-E-L-L-G-K-I-T-A-L-D-L-N-P-E-R-T-E-L-L

94 N 5 2816.430 M-E-L-L-G-K-I-T-A-L-D-L-N-P-E-R-T-E-L-L-S-C-S-R-D

95 O 5 2841.516 K-I-T-A-L-D-L-N-P-E-R-T-E-L-L-S-C-S-R-D-D-L-L-K-V

96 P 5 2913.512 D-L-N-P-E-R-T-E-L-L-S-C-S-R-D-D-L-L-K-V-I-D-L-R-T

97 Q 5 2899.580 R-T-E-L-L-S-C-S-R-D-D-L-L-K-V-I-D-L-R-T-N-A-I-K-Q

98 R 5 2790.459 S-C-S-R-D-D-L-L-K-V-I-D-L-R-T-N-A-I-K-Q-T-F-S-A-P

99 S 5 2734.473 D-L-L-K-V-I-D-L-R-T-N-A-I-K-Q-T-F-S-A-P-G-F-K-C-G

100 T 5 2811.402 I-D-L-R-T-N-A-I-K-Q-T-F-S-A-P-G-F-K-C-G-S-D-W-T-R

101 A 6 2742.348 N-A-I-K-Q-T-F-S-A-P-G-F-K-C-G-S-D-W-T-R-V-V-F-S-P

102 B 6 2709.242 T-F-S-A-P-G-F-K-C-G-S-D-W-T-R-V-V-F-S-P-D-G-S-Y-V

103 C 6 2563.169 G-F-K-C-G-S-D-W-T-R-V-V-F-S-P-D-G-S-Y-V-A-A-G-S-A

104 D 6 2620.197 S-D-W-T-R-V-V-F-S-P-D-G-S-Y-V-A-A-G-S-A-E-G-S-L-Y

105 E 6 2573.258 V-V-F-S-P-D-G-S-Y-V-A-A-G-S-A-E-G-S-L-Y-I-W-S-V-L

106 F 6 2558.243 D-G-S-Y-V-A-A-G-S-A-E-G-S-L-Y-I-W-S-V-L-T-G-K-V-E

107 G 6 2592.405 A-A-G-S-A-E-G-S-L-Y-I-W-S-V-L-T-G-K-V-E-K-V-L-S-K

108 H 6 2761.454 E-G-S-L-Y-I-W-S-V-L-T-G-K-V-E-K-V-L-S-K-Q-H-S-S-S

109 I 6 2680.480 I-W-S-V-L-T-G-K-V-E-K-V-L-S-K-Q-H-S-S-S-I-N-A-V-A

110 J 6 2596.350 T-G-K-V-E-K-V-L-S-K-Q-H-S-S-S-I-N-A-V-A-W-S-P-S-G

111 K 6 2591.335 K-V-L-S-K-Q-H-S-S-S-I-N-A-V-A-W-S-P-S-G-S-H-V-V-S

112 L 6 2538.181 Q-H-S-S-S-I-N-A-V-A-W-S-P-S-G-S-H-V-V-S-V-D-K-G-C

113 M 6 2609.331 I-N-A-V-A-W-S-P-S-G-S-H-V-V-S-V-D-K-G-C-K-A-V-L-W

114 N 6 2673.326 V-A-W-S-P-S-G-S-H-V-V-S-V-D-K-G-C-K-A-V-L-W-A-Q-Y

115 O 6 0 (space)

116 P 6 2948.474 W-S-D-N-R-A-V-Y-T-S-P-F-M-K-L-I-H-G-E-Q-L-I-E-S-K

117 Q 6 2929.465 Q-S-D-N-R-A-V-Y-T-S-P-W-M-K-L-I-H-G-E-Q-L-I-E-S-K

118 R 6 2971.490 Q-S-D-N-R-A-V-W-T-S-P-F-M-K-L-I-H-G-E-W-L-I-E-S-K

119 S 6 2948.438 Q-S-D-N-R-A-V-Y-T-S-P-F-M-K-L-I-H-G-E-Q-L-I-E-S-W

120 T 6 0 (space)

121 A 7 3067.600 M-S-S-G-L-R-A-A-D-F-P-R-W-K-R-H-I-S-E-Q-L-R-R-R-D

122 B 7 3273.793 R-A-A-D-F-P-R-W-K-R-H-I-S-E-Q-L-R-R-R-D-R-L-Q-R-Q

123 C 7 3302.797 P-R-W-K-R-H-I-S-E-Q-L-R-R-R-D-R-L-Q-R-Q-A-F-E-E-I

124 D 7 3210.701 H-I-S-E-Q-L-R-R-R-D-R-L-Q-R-Q-A-F-E-E-I-I-L-Q-Y-N

125 E 7 3227.826 L-R-R-R-D-R-L-Q-R-Q-A-F-E-E-I-I-L-Q-Y-N-K-L-L-E-K

126 F 7 3070.645 R-L-Q-R-Q-A-F-E-E-I-I-L-Q-Y-N-K-L-L-E-K-S-D-L-H-S

127 G 7 2928.585 A-F-E-E-I-I-L-Q-Y-N-K-L-L-E-K-S-D-L-H-S-V-L-A-Q-K

128 H 7 2908.628 I-L-Q-Y-N-K-L-L-E-K-S-D-L-H-S-V-L-A-Q-K-L-Q-A-E-K

129 I 7 2839.545 K-L-L-E-K-S-D-L-H-S-V-L-A-Q-K-L-Q-A-E-K-H-D-V-P-N

130 J 7 2850.463 S-D-L-H-S-V-L-A-Q-K-L-Q-A-E-K-H-D-V-P-N-R-H-E-I-S

131 K 7 2774.410 V-L-A-Q-K-L-Q-A-E-K-H-D-V-P-N-R-H-E-I-S-P-G-H-D-G

132 L 7 2865.307 L-Q-A-E-K-H-D-V-P-N-R-H-E-I-S-P-G-H-D-G-T-W-N-D-N

133 M 7 2925.274 H-D-V-P-N-R-H-E-I-S-P-G-H-D-G-T-W-N-D-N-Q-L-Q-E-M

134 N 7 2944.389 R-H-E-I-S-P-G-H-D-G-T-W-N-D-N-Q-L-Q-E-M-A-Q-L-R-I

135 O 7 2973.368 P-G-H-D-G-T-W-N-D-N-Q-L-Q-E-M-A-Q-L-R-I-K-H-Q-E-E

136 P 7 3103.503 T-W-N-D-N-Q-L-Q-E-M-A-Q-L-R-I-K-H-Q-E-E-L-T-E-L-H

137 Q 7 3071.619 Q-L-Q-E-M-A-Q-L-R-I-K-H-Q-E-E-L-T-E-L-H-K-K-R-G-E

138 R 7 2966.667 A-Q-L-R-I-K-H-Q-E-E-L-T-E-L-H-K-K-R-G-E-L-A-Q-L-V

139 S 7 2954.583 K-H-Q-E-E-L-T-E-L-H-K-K-R-G-E-L-A-Q-L-V-I-D-L-N-N

140 T 7 2974.639 L-T-E-L-H-K-K-R-G-E-L-A-Q-L-V-I-D-L-N-N-Q-M-Q-R-K

141 A 8 3040.591 K-K-R-G-E-L-A-Q-L-V-I-D-L-N-N-Q-M-Q-R-K-D-R-E-M-Q

142 B 8 3015.494 L-A-Q-L-V-I-D-L-N-N-Q-M-Q-R-K-D-R-E-M-Q-M-N-E-A-K

143 C 8 3020.419 I-D-L-N-N-Q-M-Q-R-K-D-R-E-M-Q-M-N-E-A-K-I-A-E-C-L

144 D 8 2995.387 Q-M-Q-R-K-D-R-E-M-Q-M-N-E-A-K-I-A-E-C-L-Q-T-I-S-D

145 E 8 2899.260 D-R-E-M-Q-M-N-E-A-K-I-A-E-C-L-Q-T-I-S-D-L-E-T-E-C

146 F 8 2838.334 M-N-E-A-K-I-A-E-C-L-Q-T-I-S-D-L-E-T-E-C-L-D-L-R-T

147 G 8 2837.375 I-A-E-C-L-Q-T-I-S-D-L-E-T-E-C-L-D-L-R-T-K-L-C-D-L

148 H 8 2906.400 Q-T-I-S-D-L-E-T-E-C-L-D-L-R-T-K-L-C-D-L-E-R-A-N-Q

149 I 8 2948.447 L-E-T-E-C-L-D-L-R-T-K-L-C-D-L-E-R-A-N-Q-T-L-K-D-E

150 J 8 2963.491 L-D-L-R-T-K-L-C-D-L-E-R-A-N-Q-T-L-K-D-E-Y-D-A-L-Q

151 K 8 2898.432 K-L-C-D-L-E-R-A-N-Q-T-L-K-D-E-Y-D-A-L-Q-I-T-F-T-A

152 L 8 2866.460 E-R-A-N-Q-T-L-K-D-E-Y-D-A-L-Q-I-T-F-T-A-L-E-G-K-L

153 M 8 2883.512 T-L-K-D-E-Y-D-A-L-Q-I-T-F-T-A-L-E-G-K-L-R-K-T-T-E

154 N 8 2910.486 Y-D-A-L-Q-I-T-F-T-A-L-E-G-K-L-R-K-T-T-E-E-N-Q-E-L

155 O 8 2993.553 I-T-F-T-A-L-E-G-K-L-R-K-T-T-E-E-N-Q-E-L-V-T-R-W-M

156 P 8 2987.539 L-E-G-K-L-R-K-T-T-E-E-N-Q-E-L-V-T-R-W-M-A-E-K-A-Q

157 Q 8 3030.519 R-K-T-T-E-E-N-Q-E-L-V-T-R-W-M-A-E-K-A-Q-E-A-N-R-L

158 R 8 2972.394 E-N-Q-E-L-V-T-R-W-M-A-E-K-A-Q-E-A-N-R-L-N-A-E-N-E

159 S 8 3001.479 V-T-R-W-M-A-E-K-A-Q-E-A-N-R-L-N-A-E-N-E-K-D-S-R-R

160 T 8 2952.524 A-E-K-A-Q-E-A-N-R-L-N-A-E-N-E-K-D-S-R-R-R-Q-A-R-L

161 A 9 2994.571 E-A-N-R-L-N-A-E-N-E-K-D-S-R-R-R-Q-A-R-L-Q-K-E-L-A

162 B 9 2939.517 N-A-E-N-E-K-D-S-R-R-R-Q-A-R-L-Q-K-E-L-A-E-A-A-K-E

163 C 9 2917.610 K-D-S-R-R-R-Q-A-R-L-Q-K-E-L-A-E-A-A-K-E-P-L-P-V-E

164 D 9 2861.477 R-Q-A-R-L-Q-K-E-L-A-E-A-A-K-E-P-L-P-V-E-Q-D-D-D-I

165 E 9 2792.386 Q-K-E-L-A-E-A-A-K-E-P-L-P-V-E-Q-D-D-D-I-E-V-I-V-D

166 F 9 2792.277 E-A-A-K-E-P-L-P-V-E-Q-D-D-D-I-E-V-I-V-D-E-T-S-D-H

167 G 9 2811.235 P-L-P-V-E-Q-D-D-D-I-E-V-I-V-D-E-T-S-D-H-T-E-E-T-S

168 H 9 2812.278 Q-D-D-D-I-E-V-I-V-D-E-T-S-D-H-T-E-E-T-S-P-V-R-A-I

169 I 9 2712.309 E-V-I-V-D-E-T-S-D-H-T-E-E-T-S-P-V-R-A-I-S-R-A-A-T

170 J 9 2742.353 E-T-S-D-H-T-E-E-T-S-P-V-R-A-I-S-R-A-A-T-R-R-S-V-S

171 K 9 2700.420 T-E-E-T-S-P-V-R-A-I-S-R-A-A-T-R-R-S-V-S-S-F-P-V-P

172 L 9 2724.431 P-V-R-A-I-S-R-A-A-T-R-R-S-V-S-S-F-P-V-P-Q-D-N-V-D

173 M 9 2667.300 S-R-A-A-T-R-R-S-V-S-S-F-P-V-P-Q-D-N-V-D-T-H-P-G-S

174 N 9 2750.374 R-R-S-V-S-S-F-P-V-P-Q-D-N-V-D-T-H-P-G-S-G-K-E-V-R

175 O 9 2604.282 S-F-P-V-P-Q-D-N-V-D-T-H-P-G-S-G-K-E-V-R-V-P-A-T-A

176 P 9 2654.265 Q-D-N-V-D-T-H-P-G-S-G-K-E-V-R-V-P-A-T-A-L-C-V-F-D

177 Q 9 2592.228 T-H-P-G-S-G-K-E-V-R-V-P-A-T-A-L-C-V-F-D-A-H-D-G-E

178 R 9 2624.291 G-K-E-V-R-V-P-A-T-A-L-C-V-F-D-A-H-D-G-E-V-N-A-V-Q

179 S 9 2530.169 V-P-A-T-A-L-C-V-F-D-A-H-D-G-E-V-N-A-V-Q-F-S-P-G-S

180 T 9 2645.280 L-C-V-F-D-A-H-D-G-E-V-N-A-V-Q-F-S-P-G-S-R-L-L-A-T

181 A10 2584.234 A-H-D-G-E-V-N-A-V-Q-F-S-P-G-S-R-L-L-A-T-G-G-M-D-R

182 B10 2757.475 V-N-A-V-Q-F-S-P-G-S-R-L-L-A-T-G-G-M-D-R-R-V-K-L-W

183 C10 2807.443 F-S-P-G-S-R-L-L-A-T-G-G-M-D-R-R-V-K-L-W-E-V-F-G-E

184 D10 2967.546 R-L-L-A-T-G-G-M-D-R-R-V-K-L-W-E-V-F-G-E-K-C-E-F-K

185 E10 2814.383 G-G-M-D-R-R-V-K-L-W-E-V-F-G-E-K-C-E-F-K-G-S-L-S-G

186 F10 2740.390 R-V-K-L-W-E-V-F-G-E-K-C-E-F-K-G-S-L-S-G-S-N-A-G-I

187 G10 2635.237 E-V-F-G-E-K-C-E-F-K-G-S-L-S-G-S-N-A-G-I-T-S-I-E-F

188 H10 2491.143 K-C-E-F-K-G-S-L-S-G-S-N-A-G-I-T-S-I-E-F-D-S-A-G-S

189 I10 2387.138 G-S-L-S-G-S-N-A-G-I-T-S-I-E-F-D-S-A-G-S-Y-L-L-A-A

190 J10 2520.155 S-N-A-G-I-T-S-I-E-F-D-S-A-G-S-Y-L-L-A-A-S-N-D-F-A

191 K10 2721.281 T-S-I-E-F-D-S-A-G-S-Y-L-L-A-A-S-N-D-F-A-S-R-I-W-T

192 L10 2792.293 D-S-A-G-S-Y-L-L-A-A-S-N-D-F-A-S-R-I-W-T-V-D-D-Y-R

193 M10 2995.519 Y-L-L-A-A-S-N-D-F-A-S-R-I-W-T-V-D-D-Y-R-L-R-H-T-L

194 N10 2903.395 S-N-D-F-A-S-R-I-W-T-V-D-D-Y-R-L-R-H-T-L-T-G-H-S-G

195 O10 2867.504 S-R-I-W-T-V-D-D-Y-R-L-R-H-T-L-T-G-H-S-G-K-V-L-S-A

196 P10 2840.519 V-D-D-Y-R-L-R-H-T-L-T-G-H-S-G-K-V-L-S-A-K-F-L-L-D

197 Q10 2745.566 L-R-H-T-L-T-G-H-S-G-K-V-L-S-A-K-F-L-L-D-N-A-R-I-V

198 R10 2608.361 T-G-H-S-G-K-V-L-S-A-K-F-L-L-D-N-A-R-I-V-S-G-S-H-D

199 S10 2780.591 K-V-L-S-A-K-F-L-L-D-N-A-R-I-V-S-G-S-H-D-R-T-L-K-L

200 T10 2939.598 K-F-L-L-D-N-A-R-I-V-S-G-S-H-D-R-T-L-K-L-W-D-L-R-S

201 A11 2894.591 N-A-R-I-V-S-G-S-H-D-R-T-L-K-L-W-D-L-R-S-K-V-C-I-K

202 B11 2816.501 S-G-S-H-D-R-T-L-K-L-W-D-L-R-S-K-V-C-I-K-T-V-F-A-G

203 C11 2839.473 R-T-L-K-L-W-D-L-R-S-K-V-C-I-K-T-V-F-A-G-S-S-C-N-D

204 D11 2773.313 W-D-L-R-S-K-V-C-I-K-T-V-F-A-G-S-S-C-N-D-I-V-C-T-E

205 E11 2664.198 K-V-C-I-K-T-V-F-A-G-S-S-C-N-D-I-V-C-T-E-Q-C-V-M-S

206 F11 2677.117 T-V-F-A-G-S-S-C-N-D-I-V-C-T-E-Q-C-V-M-S-G-H-F-D-K

207 G11 2932.302 S-S-C-N-D-I-V-C-T-E-Q-C-V-M-S-G-H-F-D-K-K-I-R-F-W

208 H11 3026.445 I-V-C-T-E-Q-C-V-M-S-G-H-F-D-K-K-I-R-F-W-D-I-R-S-E

209 I11 3065.522 Q-C-V-M-S-G-H-F-D-K-K-I-R-F-W-D-I-R-S-E-S-I-V-R-E

210 J11 3060.586 G-H-F-D-K-K-I-R-F-W-D-I-R-S-E-S-I-V-R-E-M-E-L-L-G

211 K11 3002.663 K-I-R-F-W-D-I-R-S-E-S-I-V-R-E-M-E-L-L-G-K-I-T-A-L

212 L11 2840.484 D-I-R-S-E-S-I-V-R-E-M-E-L-L-G-K-I-T-A-L-D-L-N-P-E

213 M11 2852.557 S-I-V-R-E-M-E-L-L-G-K-I-T-A-L-D-L-N-P-E-R-T-E-L-L

214 N11 2816.430 M-E-L-L-G-K-I-T-A-L-D-L-N-P-E-R-T-E-L-L-S-C-S-R-D

215 O11 2841.516 K-I-T-A-L-D-L-N-P-E-R-T-E-L-L-S-C-S-R-D-D-L-L-K-V

216 P11 2913.512 D-L-N-P-E-R-T-E-L-L-S-C-S-R-D-D-L-L-K-V-I-D-L-R-T

217 Q11 2899.580 R-T-E-L-L-S-C-S-R-D-D-L-L-K-V-I-D-L-R-T-N-A-I-K-Q

218 R11 2790.459 S-C-S-R-D-D-L-L-K-V-I-D-L-R-T-N-A-I-K-Q-T-F-S-A-P

219 S11 2734.473 D-L-L-K-V-I-D-L-R-T-N-A-I-K-Q-T-F-S-A-P-G-F-K-C-G

220 T11 2811.402 I-D-L-R-T-N-A-I-K-Q-T-F-S-A-P-G-F-K-C-G-S-D-W-T-R

221 A12 2742.348 N-A-I-K-Q-T-F-S-A-P-G-F-K-C-G-S-D-W-T-R-V-V-F-S-P

222 B12 2709.242 T-F-S-A-P-G-F-K-C-G-S-D-W-T-R-V-V-F-S-P-D-G-S-Y-V

223 C12 2563.169 G-F-K-C-G-S-D-W-T-R-V-V-F-S-P-D-G-S-Y-V-A-A-G-S-A

224 D12 2620.197 S-D-W-T-R-V-V-F-S-P-D-G-S-Y-V-A-A-G-S-A-E-G-S-L-Y

225 E12 2573.258 V-V-F-S-P-D-G-S-Y-V-A-A-G-S-A-E-G-S-L-Y-I-W-S-V-L

226 F12 2558.243 D-G-S-Y-V-A-A-G-S-A-E-G-S-L-Y-I-W-S-V-L-T-G-K-V-E

227 G12 2592.405 A-A-G-S-A-E-G-S-L-Y-I-W-S-V-L-T-G-K-V-E-K-V-L-S-K

228 H12 2761.454 E-G-S-L-Y-I-W-S-V-L-T-G-K-V-E-K-V-L-S-K-Q-H-S-S-S

229 I12 2680.480 I-W-S-V-L-T-G-K-V-E-K-V-L-S-K-Q-H-S-S-S-I-N-A-V-A

230 J12 2596.350 T-G-K-V-E-K-V-L-S-K-Q-H-S-S-S-I-N-A-V-A-W-S-P-S-G

231 K12 2591.335 K-V-L-S-K-Q-H-S-S-S-I-N-A-V-A-W-S-P-S-G-S-H-V-V-S

232 L12 2538.181 Q-H-S-S-S-I-N-A-V-A-W-S-P-S-G-S-H-V-V-S-V-D-K-G-C

233 M12 2609.331 I-N-A-V-A-W-S-P-S-G-S-H-V-V-S-V-D-K-G-C-K-A-V-L-W

234 N12 2673.326 V-A-W-S-P-S-G-S-H-V-V-S-V-D-K-G-C-K-A-V-L-W-A-Q-Y

235 O12 0 (space)

236 P12 2948.474 W-S-D-N-R-A-V-Y-T-S-P-F-M-K-L-I-H-G-E-Q-L-I-E-S-K

237 Q12 2929.465 Q-S-D-N-R-A-V-Y-T-S-P-W-M-K-L-I-H-G-E-Q-L-I-E-S-K

238 R12 2971.490 Q-S-D-N-R-A-V-W-T-S-P-F-M-K-L-I-H-G-E-W-L-I-E-S-K

239 S12 2948.438 Q-S-D-N-R-A-V-Y-T-S-P-F-M-K-L-I-H-G-E-Q-L-I-E-S-W

240 T12 0 (space)

ATG16L1β SPOTS MAP

**Spot Position MolWeight Sequence**

**1 A 1 3067.600 M-S-S-G-L-R-A-A-D-F-P-R-W-K-R-H-I-S-E-Q-L-R-R-R-D**

**2 B 1 3273.793 R-A-A-D-F-P-R-W-K-R-H-I-S-E-Q-L-R-R-R-D-R-L-Q-R-Q**

**3 C 1 3302.797 P-R-W-K-R-H-I-S-E-Q-L-R-R-R-D-R-L-Q-R-Q-A-F-E-E-I**

**4 D 1 3210.701 H-I-S-E-Q-L-R-R-R-D-R-L-Q-R-Q-A-F-E-E-I-I-L-Q-Y-N**

**5 E 1 3227.826 L-R-R-R-D-R-L-Q-R-Q-A-F-E-E-I-I-L-Q-Y-N-K-L-L-E-K**

**6 F 1 3070.645 R-L-Q-R-Q-A-F-E-E-I-I-L-Q-Y-N-K-L-L-E-K-S-D-L-H-S**

**7 G 1 2928.585 A-F-E-E-I-I-L-Q-Y-N-K-L-L-E-K-S-D-L-H-S-V-L-A-Q-K**

**8 H 1 2908.628 I-L-Q-Y-N-K-L-L-E-K-S-D-L-H-S-V-L-A-Q-K-L-Q-A-E-K**

**9 I 1 2839.545 K-L-L-E-K-S-D-L-H-S-V-L-A-Q-K-L-Q-A-E-K-H-D-V-P-N**

**10 J 1 2850.463 S-D-L-H-S-V-L-A-Q-K-L-Q-A-E-K-H-D-V-P-N-R-H-E-I-S**

**11 K 1 2774.410 V-L-A-Q-K-L-Q-A-E-K-H-D-V-P-N-R-H-E-I-S-P-G-H-D-G**

**12 L 1 2865.307 L-Q-A-E-K-H-D-V-P-N-R-H-E-I-S-P-G-H-D-G-T-W-N-D-N**

**13 M 1 2925.274 H-D-V-P-N-R-H-E-I-S-P-G-H-D-G-T-W-N-D-N-Q-L-Q-E-M**

**14 N 1 2944.389 R-H-E-I-S-P-G-H-D-G-T-W-N-D-N-Q-L-Q-E-M-A-Q-L-R-I**

**15 O 1 2973.368 P-G-H-D-G-T-W-N-D-N-Q-L-Q-E-M-A-Q-L-R-I-K-H-Q-E-E**

**16 P 1 3103.503 T-W-N-D-N-Q-L-Q-E-M-A-Q-L-R-I-K-H-Q-E-E-L-T-E-L-H**

**17 Q 1 3071.619 Q-L-Q-E-M-A-Q-L-R-I-K-H-Q-E-E-L-T-E-L-H-K-K-R-G-E**

**18 R 1 2966.667 A-Q-L-R-I-K-H-Q-E-E-L-T-E-L-H-K-K-R-G-E-L-A-Q-L-V**

**19 S 1 2954.583 K-H-Q-E-E-L-T-E-L-H-K-K-R-G-E-L-A-Q-L-V-I-D-L-N-N**

**20 T 1 2974.639 L-T-E-L-H-K-K-R-G-E-L-A-Q-L-V-I-D-L-N-N-Q-M-Q-R-K**

**21 A 2 3040.591 K-K-R-G-E-L-A-Q-L-V-I-D-L-N-N-Q-M-Q-R-K-D-R-E-M-Q**

**22 B 2 3015.494 L-A-Q-L-V-I-D-L-N-N-Q-M-Q-R-K-D-R-E-M-Q-M-N-E-A-K**

**23 C 2 3020.419 I-D-L-N-N-Q-M-Q-R-K-D-R-E-M-Q-M-N-E-A-K-I-A-E-C-L**

**24 D 2 2995.387 Q-M-Q-R-K-D-R-E-M-Q-M-N-E-A-K-I-A-E-C-L-Q-T-I-S-D**

**25 E 2 2899.260 D-R-E-M-Q-M-N-E-A-K-I-A-E-C-L-Q-T-I-S-D-L-E-T-E-C**

**26 F 2 2838.334 M-N-E-A-K-I-A-E-C-L-Q-T-I-S-D-L-E-T-E-C-L-D-L-R-T**

**27 G 2 2837.375 I-A-E-C-L-Q-T-I-S-D-L-E-T-E-C-L-D-L-R-T-K-L-C-D-L**

**28 H 2 2906.400 Q-T-I-S-D-L-E-T-E-C-L-D-L-R-T-K-L-C-D-L-E-R-A-N-Q**

**29 I 2 2948.447 L-E-T-E-C-L-D-L-R-T-K-L-C-D-L-E-R-A-N-Q-T-L-K-D-E**

**30 J 2 2963.491 L-D-L-R-T-K-L-C-D-L-E-R-A-N-Q-T-L-K-D-E-Y-D-A-L-Q**

**31 K 2 2898.432 K-L-C-D-L-E-R-A-N-Q-T-L-K-D-E-Y-D-A-L-Q-I-T-F-T-A**

**32 L 2 2866.460 E-R-A-N-Q-T-L-K-D-E-Y-D-A-L-Q-I-T-F-T-A-L-E-G-K-L**

**33 M 2 2883.512 T-L-K-D-E-Y-D-A-L-Q-I-T-F-T-A-L-E-G-K-L-R-K-T-T-E**

**34 N 2 2910.486 Y-D-A-L-Q-I-T-F-T-A-L-E-G-K-L-R-K-T-T-E-E-N-Q-E-L**

**35 O 2 2993.553 I-T-F-T-A-L-E-G-K-L-R-K-T-T-E-E-N-Q-E-L-V-T-R-W-M**

**36 P 2 2987.539 L-E-G-K-L-R-K-T-T-E-E-N-Q-E-L-V-T-R-W-M-A-E-K-A-Q**

**37 Q 2 3030.519 R-K-T-T-E-E-N-Q-E-L-V-T-R-W-M-A-E-K-A-Q-E-A-N-R-L**

**38 R 2 2972.394 E-N-Q-E-L-V-T-R-W-M-A-E-K-A-Q-E-A-N-R-L-N-A-E-N-E**

**39 S 2 3001.479 V-T-R-W-M-A-E-K-A-Q-E-A-N-R-L-N-A-E-N-E-K-D-S-R-R**

**40 T 2 2952.524 A-E-K-A-Q-E-A-N-R-L-N-A-E-N-E-K-D-S-R-R-R-Q-A-R-L**

**41 A 3 2994.571 E-A-N-R-L-N-A-E-N-E-K-D-S-R-R-R-Q-A-R-L-Q-K-E-L-A**

**42 B 3 2939.517 N-A-E-N-E-K-D-S-R-R-R-Q-A-R-L-Q-K-E-L-A-E-A-A-K-E**

**43 C 3 2917.610 K-D-S-R-R-R-Q-A-R-L-Q-K-E-L-A-E-A-A-K-E-P-L-P-V-E**

**44 D 3 2861.477 R-Q-A-R-L-Q-K-E-L-A-E-A-A-K-E-P-L-P-V-E-Q-D-D-D-I**

**45 E 3 2792.386 Q-K-E-L-A-E-A-A-K-E-P-L-P-V-E-Q-D-D-D-I-E-V-I-V-D**

**46 F 3 2792.277 E-A-A-K-E-P-L-P-V-E-Q-D-D-D-I-E-V-I-V-D-E-T-S-D-H**

**47 G 3 2811.235 P-L-P-V-E-Q-D-D-D-I-E-V-I-V-D-E-T-S-D-H-T-E-E-T-S**

**48 H 3 2812.278 Q-D-D-D-I-E-V-I-V-D-E-T-S-D-H-T-E-E-T-S-P-V-R-A-I**

**49 I 3 2712.309 E-V-I-V-D-E-T-S-D-H-T-E-E-T-S-P-V-R-A-I-S-R-A-A-T**

**50 J 3 2769.389 E-T-S-D-H-T-E-E-T-S-P-V-R-A-I-S-R-A-A-T-K-R-L-S-Q**

**51 K 3 2595.398 T-E-E-T-S-P-V-R-A-I-S-R-A-A-T-K-R-L-S-Q-P-A-G-G-L**

**52 L 3 2577.460 P-V-R-A-I-S-R-A-A-T-K-R-L-S-Q-P-A-G-G-L-L-D-S-I-T**

**53 M 3 2628.435 S-R-A-A-T-K-R-L-S-Q-P-A-G-G-L-L-D-S-I-T-N-I-F-G-R**

**54 N 3 2658.445 K-R-L-S-Q-P-A-G-G-L-L-D-S-I-T-N-I-F-G-R-R-S-V-S-S**

**55 O 3 2614.376 P-A-G-G-L-L-D-S-I-T-N-I-F-G-R-R-S-V-S-S-F-P-V-P-Q**

**56 P 3 2763.372 L-D-S-I-T-N-I-F-G-R-R-S-V-S-S-F-P-V-P-Q-D-N-V-D-T**

**57 Q 3 2669.284 N-I-F-G-R-R-S-V-S-S-F-P-V-P-Q-D-N-V-D-T-H-P-G-S-G**

**58 R 3 2693.341 R-S-V-S-S-F-P-V-P-Q-D-N-V-D-T-H-P-G-S-G-K-E-V-R-V**

**59 S 3 2630.334 F-P-V-P-Q-D-N-V-D-T-H-P-G-S-G-K-E-V-R-V-P-A-T-A-L**

**60 T 3 2597.243 D-N-V-D-T-H-P-G-S-G-K-E-V-R-V-P-A-T-A-L-C-V-F-D-A**

**61 A 4 2590.249 H-P-G-S-G-K-E-V-R-V-P-A-T-A-L-C-V-F-D-A-H-D-G-E-V**

**62 B 4 2714.338 K-E-V-R-V-P-A-T-A-L-C-V-F-D-A-H-D-G-E-V-N-A-V-Q-F**

**63 C 4 2587.201 P-A-T-A-L-C-V-F-D-A-H-D-G-E-V-N-A-V-Q-F-S-P-G-S-R**

**64 D 4 2589.217 C-V-F-D-A-H-D-G-E-V-N-A-V-Q-F-S-P-G-S-R-L-L-A-T-G**

**65 E 4 2669.298 H-D-G-E-V-N-A-V-Q-F-S-P-G-S-R-L-L-A-T-G-G-M-D-R-R**

**66 F 4 2787.449 N-A-V-Q-F-S-P-G-S-R-L-L-A-T-G-G-M-D-R-R-V-K-L-W-E**

**67 G 4 2788.470 S-P-G-S-R-L-L-A-T-G-G-M-D-R-R-V-K-L-W-E-V-F-G-E-K**

**68 H 4 2868.467 L-L-A-T-G-G-M-D-R-R-V-K-L-W-E-V-F-G-E-K-C-E-F-K-G**

**69 I 4 2844.394 G-M-D-R-R-V-K-L-W-E-V-F-G-E-K-C-E-F-K-G-S-L-S-G-S**

**70 J 4 2685.336 V-K-L-W-E-V-F-G-E-K-C-E-F-K-G-S-L-S-G-S-N-A-G-I-T**

**71 K 4 2621.221 V-F-G-E-K-C-E-F-K-G-S-L-S-G-S-N-A-G-I-T-S-I-E-F-D**

**72 L 4 2526.111 C-E-F-K-G-S-L-S-G-S-N-A-G-I-T-S-I-E-F-D-S-A-G-S-Y**

**73 M 4 2417.149 S-L-S-G-S-N-A-G-I-T-S-I-E-F-D-S-A-G-S-Y-L-L-A-A-S**

**74 N 4 2520.155 N-A-G-I-T-S-I-E-F-D-S-A-G-S-Y-L-L-A-A-S-N-D-F-A-S**

**75 O 4 2719.302 S-I-E-F-D-S-A-G-S-Y-L-L-A-A-S-N-D-F-A-S-R-I-W-T-V**

**76 P 4 2790.350 S-A-G-S-Y-L-L-A-A-S-N-D-F-A-S-R-I-W-T-V-D-D-Y-R-L**

**77 Q 4 2933.504 L-L-A-A-S-N-D-F-A-S-R-I-W-T-V-D-D-Y-R-L-R-H-T-L-T**

**78 R 4 2944.458 N-D-F-A-S-R-I-W-T-V-D-D-Y-R-L-R-H-T-L-T-G-H-S-G-K**

**79 S 4 2908.567 R-I-W-T-V-D-D-Y-R-L-R-H-T-L-T-G-H-S-G-K-V-L-S-A-K**

**80 T 4 2855.493 D-D-Y-R-L-R-H-T-L-T-G-H-S-G-K-V-L-S-A-K-F-L-L-D-N**

**81 A 5 2719.514 R-H-T-L-T-G-H-S-G-K-V-L-S-A-K-F-L-L-D-N-A-R-I-V-S**

**82 B 5 2663.415 G-H-S-G-K-V-L-S-A-K-F-L-L-D-N-A-R-I-V-S-G-S-H-D-R**

**83 C 5 2838.576 V-L-S-A-K-F-L-L-D-N-A-R-I-V-S-G-S-H-D-R-T-L-K-L-W**

**84 D 5 2939.598 F-L-L-D-N-A-R-I-V-S-G-S-H-D-R-T-L-K-L-W-D-L-R-S-K**

**85 E 5 2881.596 A-R-I-V-S-G-S-H-D-R-T-L-K-L-W-D-L-R-S-K-V-C-I-K-T**

**86 F 5 2816.501 G-S-H-D-R-T-L-K-L-W-D-L-R-S-K-V-C-I-K-T-V-F-A-G-S**

**87 G 5 2796.456 T-L-K-L-W-D-L-R-S-K-V-C-I-K-T-V-F-A-G-S-S-C-N-D-I**

**88 H 5 2715.292 D-L-R-S-K-V-C-I-K-T-V-F-A-G-S-S-C-N-D-I-V-C-T-E-Q**

**89 I 5 2593.124 V-C-I-K-T-V-F-A-G-S-S-C-N-D-I-V-C-T-E-Q-C-V-M-S-G**

**90 J 5 2704.164 V-F-A-G-S-S-C-N-D-I-V-C-T-E-Q-C-V-M-S-G-H-F-D-K-K**

**91 K 5 2960.297 S-C-N-D-I-V-C-T-E-Q-C-V-M-S-G-H-F-D-K-K-I-R-F-W-D**

**92 L 5 3000.393 V-C-T-E-Q-C-V-M-S-G-H-F-D-K-K-I-R-F-W-D-I-R-S-E-S**

**93 M 5 3068.504 C-V-M-S-G-H-F-D-K-K-I-R-F-W-D-I-R-S-E-S-I-V-R-E-M**

**94 N 5 3131.659 H-F-D-K-K-I-R-F-W-D-I-R-S-E-S-I-V-R-E-M-E-L-L-G-K**

**95 O 5 2989.595 I-R-F-W-D-I-R-S-E-S-I-V-R-E-M-E-L-L-G-K-I-T-A-L-D**

**96 P 5 2881.558 I-R-S-E-S-I-V-R-E-M-E-L-L-G-K-I-T-A-L-D-L-N-P-E-R**

**97 Q 5 2852.557 I-V-R-E-M-E-L-L-G-K-I-T-A-L-D-L-N-P-E-R-T-E-L-L-S**

**98 R 5 2800.417 E-L-L-G-K-I-T-A-L-D-L-N-P-E-R-T-E-L-L-S-C-S-R-D-D**

**99 S 5 2826.505 I-T-A-L-D-L-N-P-E-R-T-E-L-L-S-C-S-R-D-D-L-L-K-V-I**

**100 T 5 2912.528 L-N-P-E-R-T-E-L-L-S-C-S-R-D-D-L-L-K-V-I-D-L-R-T-N**

**101 A 6 2844.527 T-E-L-L-S-C-S-R-D-D-L-L-K-V-I-D-L-R-T-N-A-I-K-Q-T**

**102 B 6 2760.448 C-S-R-D-D-L-L-K-V-I-D-L-R-T-N-A-I-K-Q-T-F-S-A-P-G**

**103 C 6 2706.478 L-L-K-V-I-D-L-R-T-N-A-I-K-Q-T-F-S-A-P-G-F-K-C-G-S**

**104 D 6 2797.386 D-L-R-T-N-A-I-K-Q-T-F-S-A-P-G-F-K-C-G-S-D-W-T-R-V**

**105 E 6 2743.332 A-I-K-Q-T-F-S-A-P-G-F-K-C-G-S-D-W-T-R-V-V-F-S-P-D**

**106 F 6 2679.232 F-S-A-P-G-F-K-C-G-S-D-W-T-R-V-V-F-S-P-D-G-S-Y-V-A**

**107 G 6 2635.190 F-K-C-G-S-D-W-T-R-V-V-F-S-P-D-G-S-Y-V-A-A-G-S-A-E**

**108 H 6 2646.249 D-W-T-R-V-V-F-S-P-D-G-S-Y-V-A-A-G-S-A-E-G-S-L-Y-I**

**109 I 6 2575.237 V-F-S-P-D-G-S-Y-V-A-A-G-S-A-E-G-S-L-Y-I-W-S-V-L-T**

**110 J 6 2571.311 G-S-Y-V-A-A-G-S-A-E-G-S-L-Y-I-W-S-V-L-T-G-K-V-E-K**

**111 K 6 2649.427 A-G-S-A-E-G-S-L-Y-I-W-S-V-L-T-G-K-V-E-K-V-L-S-K-Q**

**112 L 6 2745.495 G-S-L-Y-I-W-S-V-L-T-G-K-V-E-K-V-L-S-K-Q-H-S-S-S-I**

**113 M 6 2753.475 W-S-V-L-T-G-K-V-E-K-V-L-S-K-Q-H-S-S-S-I-N-A-V-A-W**

**114 N 6 2582.334 G-K-V-E-K-V-L-S-K-Q-H-S-S-S-I-N-A-V-A-W-S-P-S-G-S**

**115 O 6 2562.308 V-L-S-K-Q-H-S-S-S-I-N-A-V-A-W-S-P-S-G-S-H-V-V-S-V**

**116 P 6 2538.217 H-S-S-S-I-N-A-V-A-W-S-P-S-G-S-H-V-V-S-V-D-K-G-C-K**

**117 Q 6 2567.284 N-A-V-A-W-S-P-S-G-S-H-V-V-S-V-D-K-G-C-K-A-V-L-W-A**

**118 R 6 2673.326 V-A-W-S-P-S-G-S-H-V-V-S-V-D-K-G-C-K-A-V-L-W-A-Q-Y**

**119 S 6 0 (space)**

**120 T 6 0 (space)**

**121 A 7 3067.600 M-S-S-G-L-R-A-A-D-F-P-R-W-K-R-H-I-S-E-Q-L-R-R-R-D**

**122 B 7 3273.793 R-A-A-D-F-P-R-W-K-R-H-I-S-E-Q-L-R-R-R-D-R-L-Q-R-Q**

**123 C 7 3302.797 P-R-W-K-R-H-I-S-E-Q-L-R-R-R-D-R-L-Q-R-Q-A-F-E-E-I**

**124 D 7 3210.701 H-I-S-E-Q-L-R-R-R-D-R-L-Q-R-Q-A-F-E-E-I-I-L-Q-Y-N**

**125 E 7 3227.826 L-R-R-R-D-R-L-Q-R-Q-A-F-E-E-I-I-L-Q-Y-N-K-L-L-E-K**

**126 F 7 3070.645 R-L-Q-R-Q-A-F-E-E-I-I-L-Q-Y-N-K-L-L-E-K-S-D-L-H-S**

**127 G 7 2928.585 A-F-E-E-I-I-L-Q-Y-N-K-L-L-E-K-S-D-L-H-S-V-L-A-Q-K**

**128 H 7 2908.628 I-L-Q-Y-N-K-L-L-E-K-S-D-L-H-S-V-L-A-Q-K-L-Q-A-E-K**

**129 I 7 2839.545 K-L-L-E-K-S-D-L-H-S-V-L-A-Q-K-L-Q-A-E-K-H-D-V-P-N**

**130 J 7 2850.463 S-D-L-H-S-V-L-A-Q-K-L-Q-A-E-K-H-D-V-P-N-R-H-E-I-S**

**131 K 7 2774.410 V-L-A-Q-K-L-Q-A-E-K-H-D-V-P-N-R-H-E-I-S-P-G-H-D-G**

**132 L 7 2865.307 L-Q-A-E-K-H-D-V-P-N-R-H-E-I-S-P-G-H-D-G-T-W-N-D-N**

**133 M 7 2925.274 H-D-V-P-N-R-H-E-I-S-P-G-H-D-G-T-W-N-D-N-Q-L-Q-E-M**

**134 N 7 2944.389 R-H-E-I-S-P-G-H-D-G-T-W-N-D-N-Q-L-Q-E-M-A-Q-L-R-I**

**135 O 7 2973.368 P-G-H-D-G-T-W-N-D-N-Q-L-Q-E-M-A-Q-L-R-I-K-H-Q-E-E**

**136 P 7 3103.503 T-W-N-D-N-Q-L-Q-E-M-A-Q-L-R-I-K-H-Q-E-E-L-T-E-L-H**

**137 Q 7 3071.619 Q-L-Q-E-M-A-Q-L-R-I-K-H-Q-E-E-L-T-E-L-H-K-K-R-G-E**

**138 R 7 2966.667 A-Q-L-R-I-K-H-Q-E-E-L-T-E-L-H-K-K-R-G-E-L-A-Q-L-V**

**139 S 7 2954.583 K-H-Q-E-E-L-T-E-L-H-K-K-R-G-E-L-A-Q-L-V-I-D-L-N-N**

**140 T 7 2974.639 L-T-E-L-H-K-K-R-G-E-L-A-Q-L-V-I-D-L-N-N-Q-M-Q-R-K**

**141 A 8 3040.591 K-K-R-G-E-L-A-Q-L-V-I-D-L-N-N-Q-M-Q-R-K-D-R-E-M-Q**

**142 B 8 3015.494 L-A-Q-L-V-I-D-L-N-N-Q-M-Q-R-K-D-R-E-M-Q-M-N-E-A-K**

**143 C 8 3020.419 I-D-L-N-N-Q-M-Q-R-K-D-R-E-M-Q-M-N-E-A-K-I-A-E-C-L**

**144 D 8 2995.387 Q-M-Q-R-K-D-R-E-M-Q-M-N-E-A-K-I-A-E-C-L-Q-T-I-S-D**

**145 E 8 2899.260 D-R-E-M-Q-M-N-E-A-K-I-A-E-C-L-Q-T-I-S-D-L-E-T-E-C**

**146 F 8 2838.334 M-N-E-A-K-I-A-E-C-L-Q-T-I-S-D-L-E-T-E-C-L-D-L-R-T**

**147 G 8 2837.375 I-A-E-C-L-Q-T-I-S-D-L-E-T-E-C-L-D-L-R-T-K-L-C-D-L**

**148 H 8 2906.400 Q-T-I-S-D-L-E-T-E-C-L-D-L-R-T-K-L-C-D-L-E-R-A-N-Q**

**149 I 8 2948.447 L-E-T-E-C-L-D-L-R-T-K-L-C-D-L-E-R-A-N-Q-T-L-K-D-E**

**150 J 8 2963.491 L-D-L-R-T-K-L-C-D-L-E-R-A-N-Q-T-L-K-D-E-Y-D-A-L-Q**

**151 K 8 2898.432 K-L-C-D-L-E-R-A-N-Q-T-L-K-D-E-Y-D-A-L-Q-I-T-F-T-A**

**152 L 8 2866.460 E-R-A-N-Q-T-L-K-D-E-Y-D-A-L-Q-I-T-F-T-A-L-E-G-K-L**

**153 M 8 2883.512 T-L-K-D-E-Y-D-A-L-Q-I-T-F-T-A-L-E-G-K-L-R-K-T-T-E**

**154 N 8 2910.486 Y-D-A-L-Q-I-T-F-T-A-L-E-G-K-L-R-K-T-T-E-E-N-Q-E-L**

**155 O 8 2993.553 I-T-F-T-A-L-E-G-K-L-R-K-T-T-E-E-N-Q-E-L-V-T-R-W-M**

**156 P 8 2987.539 L-E-G-K-L-R-K-T-T-E-E-N-Q-E-L-V-T-R-W-M-A-E-K-A-Q**

**157 Q 8 3030.519 R-K-T-T-E-E-N-Q-E-L-V-T-R-W-M-A-E-K-A-Q-E-A-N-R-L**

**158 R 8 2972.394 E-N-Q-E-L-V-T-R-W-M-A-E-K-A-Q-E-A-N-R-L-N-A-E-N-E**

**159 S 8 3001.479 V-T-R-W-M-A-E-K-A-Q-E-A-N-R-L-N-A-E-N-E-K-D-S-R-R**

**160 T 8 2952.524 A-E-K-A-Q-E-A-N-R-L-N-A-E-N-E-K-D-S-R-R-R-Q-A-R-L**

**161 A 9 2994.571 E-A-N-R-L-N-A-E-N-E-K-D-S-R-R-R-Q-A-R-L-Q-K-E-L-A**

**162 B 9 2939.517 N-A-E-N-E-K-D-S-R-R-R-Q-A-R-L-Q-K-E-L-A-E-A-A-K-E**

**163 C 9 2917.610 K-D-S-R-R-R-Q-A-R-L-Q-K-E-L-A-E-A-A-K-E-P-L-P-V-E**

**164 D 9 2861.477 R-Q-A-R-L-Q-K-E-L-A-E-A-A-K-E-P-L-P-V-E-Q-D-D-D-I**

**165 E 9 2792.386 Q-K-E-L-A-E-A-A-K-E-P-L-P-V-E-Q-D-D-D-I-E-V-I-V-D**

**166 F 9 2792.277 E-A-A-K-E-P-L-P-V-E-Q-D-D-D-I-E-V-I-V-D-E-T-S-D-H**

**167 G 9 2811.235 P-L-P-V-E-Q-D-D-D-I-E-V-I-V-D-E-T-S-D-H-T-E-E-T-S**

**168 H 9 2812.278 Q-D-D-D-I-E-V-I-V-D-E-T-S-D-H-T-E-E-T-S-P-V-R-A-I**

**169 I 9 2712.309 E-V-I-V-D-E-T-S-D-H-T-E-E-T-S-P-V-R-A-I-S-R-A-A-T**

**170 J 9 2769.389 E-T-S-D-H-T-E-E-T-S-P-V-R-A-I-S-R-A-A-T-K-R-L-S-Q**

**171 K 9 2595.398 T-E-E-T-S-P-V-R-A-I-S-R-A-A-T-K-R-L-S-Q-P-A-G-G-L**

**172 L 9 2577.460 P-V-R-A-I-S-R-A-A-T-K-R-L-S-Q-P-A-G-G-L-L-D-S-I-T**

**173 M 9 2628.435 S-R-A-A-T-K-R-L-S-Q-P-A-G-G-L-L-D-S-I-T-N-I-F-G-R**

**174 N 9 2658.445 K-R-L-S-Q-P-A-G-G-L-L-D-S-I-T-N-I-F-G-R-R-S-V-S-S**

**175 O 9 2614.376 P-A-G-G-L-L-D-S-I-T-N-I-F-G-R-R-S-V-S-S-F-P-V-P-Q**

**176 P 9 2763.372 L-D-S-I-T-N-I-F-G-R-R-S-V-S-S-F-P-V-P-Q-D-N-V-D-T**

**177 Q 9 2669.284 N-I-F-G-R-R-S-V-S-S-F-P-V-P-Q-D-N-V-D-T-H-P-G-S-G**

**178 R 9 2693.341 R-S-V-S-S-F-P-V-P-Q-D-N-V-D-T-H-P-G-S-G-K-E-V-R-V**

**179 S 9 2630.334 F-P-V-P-Q-D-N-V-D-T-H-P-G-S-G-K-E-V-R-V-P-A-T-A-L**

**180 T 9 2597.243 D-N-V-D-T-H-P-G-S-G-K-E-V-R-V-P-A-T-A-L-C-V-F-D-A**

**181 A10 2590.249 H-P-G-S-G-K-E-V-R-V-P-A-T-A-L-C-V-F-D-A-H-D-G-E-V**

**182 B10 2714.338 K-E-V-R-V-P-A-T-A-L-C-V-F-D-A-H-D-G-E-V-N-A-V-Q-F**

**183 C10 2587.201 P-A-T-A-L-C-V-F-D-A-H-D-G-E-V-N-A-V-Q-F-S-P-G-S-R**

**184 D10 2589.217 C-V-F-D-A-H-D-G-E-V-N-A-V-Q-F-S-P-G-S-R-L-L-A-T-G**

**185 E10 2669.298 H-D-G-E-V-N-A-V-Q-F-S-P-G-S-R-L-L-A-T-G-G-M-D-R-R**

**186 F10 2787.449 N-A-V-Q-F-S-P-G-S-R-L-L-A-T-G-G-M-D-R-R-V-K-L-W-E**

**187 G10 2788.470 S-P-G-S-R-L-L-A-T-G-G-M-D-R-R-V-K-L-W-E-V-F-G-E-K**

**188 H10 2868.467 L-L-A-T-G-G-M-D-R-R-V-K-L-W-E-V-F-G-E-K-C-E-F-K-G**

**189 I10 2844.394 G-M-D-R-R-V-K-L-W-E-V-F-G-E-K-C-E-F-K-G-S-L-S-G-S**

**190 J10 2685.336 V-K-L-W-E-V-F-G-E-K-C-E-F-K-G-S-L-S-G-S-N-A-G-I-T**

**191 K10 2621.221 V-F-G-E-K-C-E-F-K-G-S-L-S-G-S-N-A-G-I-T-S-I-E-F-D**

**192 L10 2526.111 C-E-F-K-G-S-L-S-G-S-N-A-G-I-T-S-I-E-F-D-S-A-G-S-Y**

**193 M10 2417.149 S-L-S-G-S-N-A-G-I-T-S-I-E-F-D-S-A-G-S-Y-L-L-A-A-S**

**194 N10 2520.155 N-A-G-I-T-S-I-E-F-D-S-A-G-S-Y-L-L-A-A-S-N-D-F-A-S**

**195 O10 2719.302 S-I-E-F-D-S-A-G-S-Y-L-L-A-A-S-N-D-F-A-S-R-I-W-T-V**

**196 P10 2790.350 S-A-G-S-Y-L-L-A-A-S-N-D-F-A-S-R-I-W-T-V-D-D-Y-R-L**

**197 Q10 2933.504 L-L-A-A-S-N-D-F-A-S-R-I-W-T-V-D-D-Y-R-L-R-H-T-L-T**

**198 R10 2944.458 N-D-F-A-S-R-I-W-T-V-D-D-Y-R-L-R-H-T-L-T-G-H-S-G-K**

**199 S10 2908.567 R-I-W-T-V-D-D-Y-R-L-R-H-T-L-T-G-H-S-G-K-V-L-S-A-K**

**200 T10 2855.493 D-D-Y-R-L-R-H-T-L-T-G-H-S-G-K-V-L-S-A-K-F-L-L-D-N**

**201 A11 2719.514 R-H-T-L-T-G-H-S-G-K-V-L-S-A-K-F-L-L-D-N-A-R-I-V-S**

**202 B11 2663.415 G-H-S-G-K-V-L-S-A-K-F-L-L-D-N-A-R-I-V-S-G-S-H-D-R**

**203 C11 2838.576 V-L-S-A-K-F-L-L-D-N-A-R-I-V-S-G-S-H-D-R-T-L-K-L-W**

**204 D11 2939.598 F-L-L-D-N-A-R-I-V-S-G-S-H-D-R-T-L-K-L-W-D-L-R-S-K**

**205 E11 2881.596 A-R-I-V-S-G-S-H-D-R-T-L-K-L-W-D-L-R-S-K-V-C-I-K-T**

**206 F11 2816.501 G-S-H-D-R-T-L-K-L-W-D-L-R-S-K-V-C-I-K-T-V-F-A-G-S**

**207 G11 2796.456 T-L-K-L-W-D-L-R-S-K-V-C-I-K-T-V-F-A-G-S-S-C-N-D-I**

**208 H11 2715.292 D-L-R-S-K-V-C-I-K-T-V-F-A-G-S-S-C-N-D-I-V-C-T-E-Q**

**209 I11 2593.124 V-C-I-K-T-V-F-A-G-S-S-C-N-D-I-V-C-T-E-Q-C-V-M-S-G**

**210 J11 2704.164 V-F-A-G-S-S-C-N-D-I-V-C-T-E-Q-C-V-M-S-G-H-F-D-K-K**

**211 K11 2960.297 S-C-N-D-I-V-C-T-E-Q-C-V-M-S-G-H-F-D-K-K-I-R-F-W-D**

**212 L11 3000.393 V-C-T-E-Q-C-V-M-S-G-H-F-D-K-K-I-R-F-W-D-I-R-S-E-S**

**213 M11 3068.504 C-V-M-S-G-H-F-D-K-K-I-R-F-W-D-I-R-S-E-S-I-V-R-E-M**

**214 N11 3131.659 H-F-D-K-K-I-R-F-W-D-I-R-S-E-S-I-V-R-E-M-E-L-L-G-K**

**215 O11 2989.595 I-R-F-W-D-I-R-S-E-S-I-V-R-E-M-E-L-L-G-K-I-T-A-L-D**

**216 P11 2881.558 I-R-S-E-S-I-V-R-E-M-E-L-L-G-K-I-T-A-L-D-L-N-P-E-R**

**217 Q11 2852.557 I-V-R-E-M-E-L-L-G-K-I-T-A-L-D-L-N-P-E-R-T-E-L-L-S**

**218 R11 2800.417 E-L-L-G-K-I-T-A-L-D-L-N-P-E-R-T-E-L-L-S-C-S-R-D-D**

**219 S11 2826.505 I-T-A-L-D-L-N-P-E-R-T-E-L-L-S-C-S-R-D-D-L-L-K-V-I**

**220 T11 2912.528 L-N-P-E-R-T-E-L-L-S-C-S-R-D-D-L-L-K-V-I-D-L-R-T-N**

**221 A12 2844.527 T-E-L-L-S-C-S-R-D-D-L-L-K-V-I-D-L-R-T-N-A-I-K-Q-T**

**222 B12 2760.448 C-S-R-D-D-L-L-K-V-I-D-L-R-T-N-A-I-K-Q-T-F-S-A-P-G**

**223 C12 2706.478 L-L-K-V-I-D-L-R-T-N-A-I-K-Q-T-F-S-A-P-G-F-K-C-G-S**

**224 D12 2797.386 D-L-R-T-N-A-I-K-Q-T-F-S-A-P-G-F-K-C-G-S-D-W-T-R-V**

**225 E12 2743.332 A-I-K-Q-T-F-S-A-P-G-F-K-C-G-S-D-W-T-R-V-V-F-S-P-D**

**226 F12 2679.232 F-S-A-P-G-F-K-C-G-S-D-W-T-R-V-V-F-S-P-D-G-S-Y-V-A**

**227 G12 2635.190 F-K-C-G-S-D-W-T-R-V-V-F-S-P-D-G-S-Y-V-A-A-G-S-A-E**

**228 H12 2646.249 D-W-T-R-V-V-F-S-P-D-G-S-Y-V-A-A-G-S-A-E-G-S-L-Y-I**

**229 I12 2575.237 V-F-S-P-D-G-S-Y-V-A-A-G-S-A-E-G-S-L-Y-I-W-S-V-L-T**

**230 J12 2571.311 G-S-Y-V-A-A-G-S-A-E-G-S-L-Y-I-W-S-V-L-T-G-K-V-E-K**

**231 K12 2649.427 A-G-S-A-E-G-S-L-Y-I-W-S-V-L-T-G-K-V-E-K-V-L-S-K-Q**

**232 L12 2745.495 G-S-L-Y-I-W-S-V-L-T-G-K-V-E-K-V-L-S-K-Q-H-S-S-S-I**

**233 M12 2753.475 W-S-V-L-T-G-K-V-E-K-V-L-S-K-Q-H-S-S-S-I-N-A-V-A-W**

**234 N12 2582.334 G-K-V-E-K-V-L-S-K-Q-H-S-S-S-I-N-A-V-A-W-S-P-S-G-S**

**235 O12 2562.308 V-L-S-K-Q-H-S-S-S-I-N-A-V-A-W-S-P-S-G-S-H-V-V-S-V**

**236 P12 2538.217 H-S-S-S-I-N-A-V-A-W-S-P-S-G-S-H-V-V-S-V-D-K-G-C-K**

**237 Q12 2567.284 N-A-V-A-W-S-P-S-G-S-H-V-V-S-V-D-K-G-C-K-A-V-L-W-A**

**238 R12 2673.326 V-A-W-S-P-S-G-S-H-V-V-S-V-D-K-G-C-K-A-V-L-W-A-Q-Y**

**239 S12 0 (space)**

**240 T12 0 (space)**
